# Network Neurodegeneration in Alzheimer’s Disease via MRI Based Shape Diffeomorphometry and High-Field Atlasing

**DOI:** 10.3389/fbioe.2015.00054

**Published:** 2015-05-15

**Authors:** Michael I. Miller, J. Tilak Ratnanather, Daniel J. Tward, Timothy Brown, David S. Lee, Michael Ketcha, Kanami Mori, Mei-Cheng Wang, Susumu Mori, Marilyn S. Albert, Laurent Younes, BIOCARD Research Team

**Affiliations:** ^1^Center for Imaging Science, Johns Hopkins University, Baltimore, MD, USA; ^2^Institute for Computational Medicine, Johns Hopkins University, Baltimore, MD, USA; ^3^Department of Biomedical Engineering, Johns Hopkins University, Baltimore, MD, USA; ^4^Department of Biostatistics, Bloomberg School of Public Health, Johns Hopkins University, Baltimore, MD, USA; ^5^Department of Radiology, Johns Hopkins University School of Medicine, Baltimore, MD, USA; ^6^Department of Neurology, Johns Hopkins University School of Medicine, Baltimore, MD, USA; ^7^Department of Applied Mathematics and Statistics, Johns Hopkins University, Baltimore, MD, USA

**Keywords:** shape, diffeomorphometry, preclinical Alzheimer’s disease, entorhinal cortex, cell–cell hypothesis

## Abstract

This paper examines MRI analysis of neurodegeneration in Alzheimer’s Disease (AD) in a network of structures within the medial temporal lobe using diffeomorphometry methods coupled with high-field atlasing in which the entorhinal cortex is partitioned into eight subareas. The morphometry markers for three groups of subjects (controls, preclinical AD, and symptomatic AD) are indexed to template coordinates measured with respect to these eight subareas. The location and timing of changes are examined within the subareas as it pertains to the classic Braak and Braak staging by comparing the three groups. We demonstrate that the earliest preclinical changes in the population occur in the lateral most sulcal extent in the entorhinal cortex (alluded to as transentorhinal cortex by Braak and Braak), and then proceeds medially which is consistent with the Braak and Braak staging. We use high-field 11T atlasing to demonstrate that the network changes are occurring at the junctures of the substructures in this medial temporal lobe network. Temporal progression of the disease through the network is also examined via changepoint analysis, demonstrating earliest changes in entorhinal cortex. The differential expression of rate of atrophy with progression signaling the changepoint time across the network is demonstrated to be signaling in the intermediate caudal subarea of the entorhinal cortex, which has been noted to be proximal to the hippocampus. This coupled to the findings of the nearby basolateral involvement in amygdala demonstrates the selectivity of neurodegeneration in early AD.

## Introduction

Structural brain imaging via magnetic resonance imaging (MRI) has advanced our knowledge of regional brain atrophy in several major neurodegenerative brain diseases including Alzheimer’s Disease (AD). There is a consensus that MRI measures are an indirect reflection of neuronal injury occurring in the brain as AD progresses.

The three regions of the medial temporal lobe – amygdala, hippocampus, and entorhinal cortex (ERC) – are central to the examination of AD since the first histopathological findings suggest they are affected during the earliest phases of AD (Herzog and Kemper, 1980; Tsuchiya and Kosaka, 1990; Arnold et al., 1991; Scott et al., 1991, 1992; Arriagada et al., 1992). Specifically fibrillary lesions such as plaques and tangles have been observed to accumulate in these regions. The density of these lesions correlates with AD severity and may be associated with the integrity of the performant pathway (Garcia-Sierra et al., 2000). Within the medial temporal lobe, the distribution of these lesions follows a progression first beginning in layer II of the ERC, hippocampal region, CA1/subiculum connections with layer IV of the ERC, and then cortex, as first described by Braak and Braak (1991).

For at least two decades, MRI research in AD focused on patients with AD dementia and mild cognitive impairment (MCI) [for reviews see Atiya et al. (2003) and Kantarci and Jack (2004)]. More recently, attention has turned to the preclinical phase of AD, the phase when individuals are cognitively normal but pathology is accumulating. Brain changes during this early phase of AD are likely to be subtle, requiring more novel approaches for explication. Over the past decade, comparative morphology methods derivative of D’Arcy Thompson have emerged based on diffeomorphic mapping of populations to dense template coordinate systems. The introduction of diffeomorphic mapping allows for the calculation of metric change, termed *diffeomorphometry* (Miller et al., 2014b). Diffeomorphometry provides correspondences between the population and template via *geodesic positioning*, as well as to other coordinate systems such as the high field ERC and hippocampus atlases which we introduce here to study atrophy. The geodesic positioning system also provides an associated set of geodesic coordinates positioning the population in the metric space centered on the template providing a powerful statistical frame (Miller et al., 2014b). These geodesic coordinates are of the proper dimension for encoding the anatomical phenotype of the network and act as a sensitive marker of neurodegeneration. We generate population statistics on these geodesic coordinates using linear effects modeling in which significance is assessed via permutation testing against the null hypothesis while taking multiple comparisons into account.

Focusing on networks of structures via time series of biomarkers offers an opportunity to examine both the temporal onset of morphometric changes through their differential expression across the subcortical structures, as well as their differential spatial expression. We call the first our temporal ordering or changepoint progression model in which atrophy across the population is modeled as following one regimen in the control group during aging, and changes to a second regimen at some random changepoint time. The changepoint signals a dramatic shift in behavior during progression of network neurodegeneration, serving as an epoch time during one’s transition from normal morphometric change to the disease phase. The longitudinal nature of the changepoint model provides us with the progression of the groups of subjects within a common population allowing us to provide a picture into the temporal ordering of the disease, the *when* associated to the structural process of neurodegeneration. The second technology associated with geodesic positioning of the high-field atlases for the ERC and temporal lobe structures allows us to examine more carefully the *where* of the differential expression of neurodegeneration within the networks of structures. Importantly, the temporal and positional positioning technologies for studying AD allow us to explore in the living human being one of the questions associated with several neurodegenerative illnesses, the degree to which pathogenesis spreads in a systematic way within a network, and whether this spread might be consistent with cell-to-cell circuit based interactions.

## Materials and Methods

### Data

The study known as the BIOCARD study is uniquely positioned to provide information concerning the evolution of brain changes during the earliest phase of AD. All subjects were cognitively normal when recruited, their mean age at baseline was 57.1 years, and have now been followed for up to 17 years. By design, approximately three quarters of the participants had a first degree relative with dementia of the Alzheimer type. MRI scans, cerebrospinal fluid (CSF), and blood specimens were obtained every 2 years. The study was initiated at the NIH in 1995, and was stopped in 2005. In 2009, a research team at the Johns Hopkins School of Medicine was funded to re-establish the cohort, continue the annual clinical and cognitive assessments, collect blood, and evaluate the previously acquired MRI scans, CSF, and blood specimens.

The clinical and cognitive assessments of the participants have been described elsewhere (Albert et al., 2014) and only summarized here. Briefly, the cognitive assessment consisted of a neuropsychological battery covering all major cognitive domains (i.e., memory, executive function, language, spatial ability, attention, and processing speed). A clinical assessment was also conducted annually. Since the study has been conducted at Johns Hopkins, this has included the following: a physical and neurological examination, record of medication use, behavioral and mood assessments (Yesavage et al., 1982; Cummings et al., 1994), family history of dementia, history of symptom onset, and a clinical dementia rating (CDR), based on a semi-structured interview (Hughes et al., 1982; Morris, 1993).

The diagnostic procedures are comparable to those used in the Alzheimer’s Disease Research Centers program involving a two-step process by which a decision is first made about whether the subject is normal, mildly impaired, or demented (based on the clinical history and the cognitive testing), and then (if the subject is judged not to be normal) the likely cause(s) of the cognitive impairment is determined. The estimated age-of-onset of clinical symptoms used in the changepoint analyses was established during the clinical interview by the clinician, who evaluated the subject (or on the basis of clinical notes in the record) and re-confirmed during the consensus conference.

The MRI scans analyzed here were acquired during the period 1995–2005 involved 335 participants at baseline, with a total of a total of 805 obtained in total over subsequent years, with a mean of 2.3 scans per person. The mean interval between follow-up scans was 2.02 years. The scans acquired at the NIH were obtained using a standard multi-modal protocol using GE 1.5T scanner. Coronal SPGR scans were used for analyses presented here, the Coronal SPGR (Spoiled Gradient Echo) sequence (TR = 24, TE = 2, FOV = 256 × 256, thickness/gap = 2.0/0.0 mm, flip angle = 20, 124 slices).

Of the 335 subjects with scans at baseline, a total of 230 individuals remained cognitively normal, and 50 or so developed incident cognitive impairment and were diagnosed with MCI (of these, 8 subsequently progressed to AD dementia). The subjects who were control at the time of the scans were obtained but became impaired over time are referred to here as having “preclinical AD” (Sperling et al., 2011). Of the 230 control, who remained control on follow-up, 136 had repeat MRI scans (*M* = 2.98/subject). Of the 50 or so participants with preclinical AD, 33 had repeat MRI scans (*M* = 2.94/subject). In addition, 20 or so participants received a diagnosis of MCI or AD dementia during the time that the MRI scans were obtained and are referred to as the symptomatic group. The analyses described below have been completed for the amygdala, hippocampus, and ERC for 221 control subjects, 50 preclinical subjects, and 20 symptomatic subjects with average ages at entry of 55, 62, and 64, respectively, with ApoE-carrier status of 32, 33, and 70%, respectively. The number of scans within each group was 2.2, 2.3, and 3.6, respectively. For details see Miller et al. (2013).

### Diffeomorphometry and geodesic positioning

#### Surface LDDMM

Shape diffeomorphometry of subcortical structures (Miller et al., 2014a, 2013; Tang et al., 2014; Younes et al., 2014a) follows three steps: (i) segmentation of the target structures, (ii) generation of a single template coordinate system from the population of baseline scans, and (iii) generation of the shape markers by mapping of the template onto each of the target segmented structures represented via triangulated meshes. For the segmentation of the amygdala, ERC, and hippocampus structures, we use large deformation diffeomorphic metric mapping (LDDMM) with landmarks to ensure consistency of mappings (Miller et al., 2013; Younes et al., 2014a). All surfaces are rigidly aligned via rotation and translation, with right subvolumes flipped before alignment to ensure that all structures may be compared. From these sets of surfaces, we do template estimation and the linear mixed-effects (LME) modeling.

**Figure 1:**
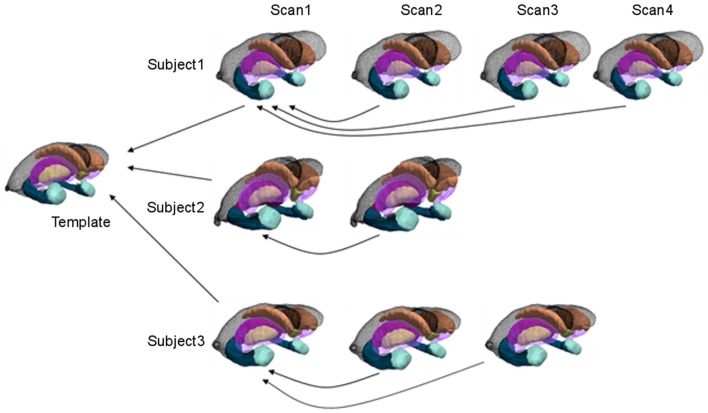
**Cross-sectional and longitudinal template-centered analyses**. For cross-sectional analyses, only first scan is depicted being carried to common template coordinates. For high-dimensional shape diffeomorphometry, the common template coordinate system is generated to which all information in the population is registered. For longitudinal analysis, template-centered analyses map every element to the template; geodesic analyses uses the subjects own scan as its subject-dependent template (within individual), from which rates of change and intercept can be computed (>2 scans for rate). For high-dimensional shape, this information is mapped cross-sectionally to the common template coordinates.

The morphometry shape statistics are indexed to a common template coordinate system by computing a smooth, invertible, diffeomorphic correspondence between the template and the surfaces using LDDMM (Vaillant et al., 2007; Qiu and Miller, 2008). As depicted in Figure 1 for the longitudinal mapping, given targets $S_{t_{1}},\;S_{t_{2}},\;\ldots ,\;S_{t_{N}},\;0<t_{1}\ldots <t_{N}\;=\;1,$ the mapping solves a variational problem transforming the triangulated surface template onto the targets satisfying ${\dot{\phi }}_{t}\;=\;v_{t}\;\circ \;\varphi_{t},\;\varphi_{\text{0}}\;=\text{ id},$ minimizing an integrated energy density ${∥v_{t}∥}_{V}^{2}$ for the vector fields, and a summed matching cost *E*(⋅,⋅) between template and surfaces given by

(1) $$\int_{0}^{1}{∥v_{t}∥}_{V}^{2}\text{dt+}\sum_{i=1}^{N}E\left(\varphi_{t_{i}}\left(S_{\mathrm{temp}}\right),S_{i}\right)$$

The first term is a geodesic distance in shape space corresponding to a least-deformation path for deforming coordinate systems (Grenander and Miller, 2007; Younes, 2010). When there is only one target surface, then the sum reduces to one term.

The *N* error terms compute the mismatch between surfaces by assuming the deformed template and target surfaces have local parameterizations $S=q\left(u\right),\;u\in U,\;S^{\prime }=q^{\prime }\left(u\right),u\in U,$ with the distance between smooth coordinates based on the disparity between the normals of the surfaces. This is described in Appendix 1.

Since the vector space *v* ∈ *V* of vector fields is spatially smooth, it has a reproducing kernel defined as *K* implying that the variational minimizers of Eq. 1 will involve the kernel (see Appendix 1). The variational problem of Eq. 1 is solved by representing the deforming surfaces as a dynamical system, with state *q*_0_, *u* ∈ *U*, *q_t_* = φ*_t_*(*S*_temp_), *q*_0_ = *S*_temp_, which evolve to match the boundary conditions given by the terms in the variational problem Eq. 1. The target surfaces enter through boundary conditions involving the state transforming the template (see Appendix 1).

#### Template Estimation from Populations

**Figure 2:**
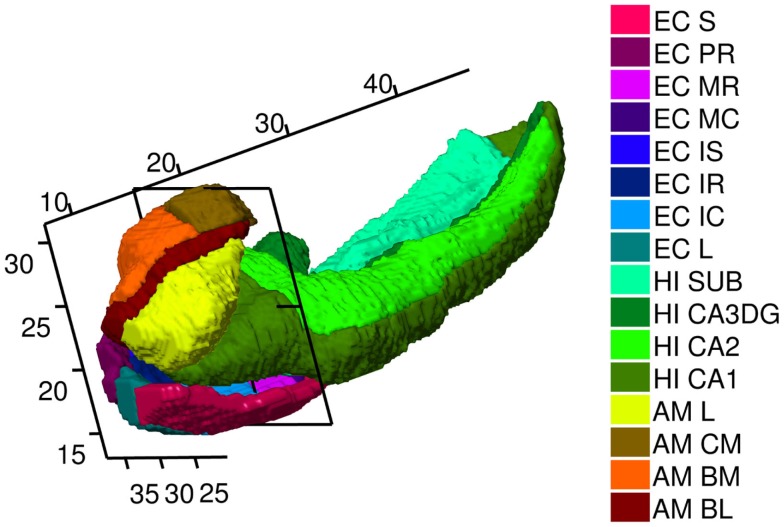
**High-field 11T atlas of amygdala, ERC, and hippocampus**. ERC parcelation as defined (Krimer et al., 1997). Abbreviations in the legend are named (from top to bottom) Sulcal, PR Prorhinal, MR, MC, Medial rostral, and caudal, IS, IC, IR intermediate superior, caudal, rostral, L lateral. Hippocampus parcelation Sub Subiculum, CA3/Dentate Gyrus, CA2, CA1. Amygdala as defined (Miller et al., 2014a) with AM, CM, BM, BL, L centromedial, basomedial basolateral, lateral, not available to this view. Planar section cuts perpendicularly at 17 mm along the rostral–caudal axis (R–C) of hippocampus through the Intermedial caudal parcel (light blue), which is most proximate to hippocampus. In the coordinate system for the temporal lobe, the R–C axis lying along the hippocampus has the rostral end of the ERC with beginning of S, L, PR, and rostral end of amygdala with lateral and basolateral structures beginning at 7.5 mm along the R–C axis. The head of the hippocampus CA1 begins caudally several mm at the 11 mm R–C axis.

While single volume numbers can be averaged across subjects, the morphometry markers must be synchronized by building correspondences across the population, requiring registration to the common template coordinates. This is performed by rigidly aligning volumes and creating a single family of template shapes using a Bayesian generative model of the surfaces as random deformations of an unknown, to be estimated, template (Ma et al., 2010). Figure 10 shows the amygdala, ERC, and hippocampus templates used for the statistical studies with the triangulated meshes with morphometry markers superimposed. The template shape coordinates were computed by running the template generation algorithm on the population of 325 baseline scans and are blind to group labels. The high-field templates shown in Figure 2 are defined with the same definitions at the high resolution as the definitions used for constructing the template in the population.

#### High Field Atlasing via Diffeomorphometry and Geodesic Positioning

Diffeomorphometry provides a geodesic positioning system enabling both the positioning or transfer of label maps from one coordinate system relative to another, as well as geodesic coordinates (Miller et al., 2014b). The geodesic coordinates encode the shape phenotype and form the biomarker Jacobian in the statistical shape analysis described below. The geodesic positioning is what is required for using our high field 11T label maps for amygdala, ERC, and hippocampus. The geodesic positioning algorithm provides diffeomorphic correspondence between the multiple atlas coordinate systems $X_{11T}\overset{\varphi }{\underset{\varphi^{-1}}{⇄}}X_{\text{biocard}}$, with the correspondence φ transporting the label maps on the high-field 11T atlas *L*_11T_(*x*), *x* ∈ *X*_11T_ from the high-definition coordinate system to the population template according to

(2) $$L_{\mathrm{biocard}}\left(x\right)=\varphi \cdot L_{11T}\left(x\right)=L_{11T}\circ \varphi^{-1}\left(x\right),\;x\in X_{11T}.$$

This equation is the algebraic definition of transporting the dense label map from one coordinate system to another, with interpolation to coordinate centers.

Figure 2 shows the high field partitions of amygdala, ERC, and the hippocampus. We have already published the high-field amygdala partition into core and non-core structures including subvolumes of lateral, basolateral, basomedial, and centromedial (Miller et al., 2014a), and a high-field hippocampus partition into CA1, CA2, CA3/dentate-gyrus, subiculum (Tang et al., 2014). Definitions for these atlases are described in http://caportal.cis.jhu.edu/protocols. Also shown in Figure 2 is the parcelation of the ERC based on eight subfields defined by Krimer et al. (1997). Five main subfields (prorhinal, lateral, intermediate, sulcal, and medial – Pr, L, I, M, and S) were defined with subareas within these subfields demarcated, with subfield I divided further into three subareas, and M and S each divided into rostral and caudal subareas. The parcelation was based on stereological measurements from post-mortem human data such as neuronal size and density as well as subdivisional volume and laminar thickness, which are different in the subfields.

Using Seg3D (Scientific Computing and Imaging Institute), the procedure for delineation begins with the ERC reconstructions (Miller et al., 2013) with the ERC viewed as the representation of the Brodmann Area 28 and part of Area 35. Then the six coronal sections that appear visually correspondent to the sections in Figures 3A–F of Krimer et al. (1997) were located. These sections are effectively the lines A–F delineated in the 2D parcelation map of Van Essen and Maunsell (1980) into the five main subfields Pr, L, I, M, and S; see Figure 2 in Krimer et al. (1997). In each of the six sections, landmarks corresponding to the lateral and medial extents of the subfields were placed, with the landmarks joined by lines along the pial and gray/white surfaces. Appendix 4 describes these subfields. In Figure 2, the axis coordinate system is shown in millimeters, with the rostral–caudal (R–C) axis in parallel with that of the hippocampus. The planar section is shown being perpendicular to the R–C axis through the intermedial caudal partition (light blue) at 17 mm along the R–C axis. In our coordinate system, the R–C axis lying along the hippocampus has the rostral end of the ERC with beginning of S, L, PR, and rostral end of amygdala with lateral and basolateral begin at 7.5 mm along the R–C axis. The head of the hippocampal CA1 subfield begins caudally at the 11 mm point of the R–C axis.

**Figure 3:**
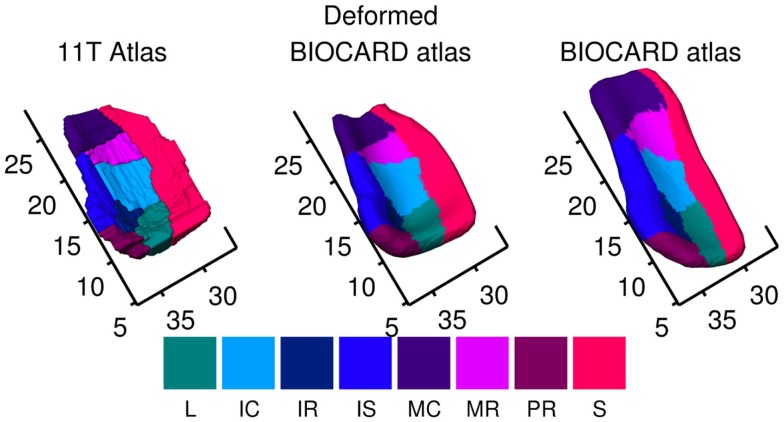
**Mapping of labels from high-field 11T ERC atlas onto BIOCARD ERC population template showing local coordinate system in millimeters around the ERC**. Column 1 shows high field parcelation of 11T atlas; column 2 shows BIOCARD population atlas deformed onto the 11T atlas transferring 11T high field subfield labels; column 3 shows high-field atlas labels transferred to BIOCARD template. The regions abbreviated in the legend are named (from top to bottom) Sulcal, Prorhinal, Medial rostral, Medial caudal, Intermediate superior, Intermediate rostral, Intermediate caudal, Lateral. S in dark red denotes sulcal region which is most lateral. Biocard (right column) has been registered to high-field atlas (left column); so rostral–caudal axis has units in millimeters associated to Figure 2, 11T atlas. ERC starts at 7.5 mm rostral–caudal.

Figure 3 shows mapping of labels from the high field 11T ERC atlas onto BIOCARD ERC population template Eq. 3a. Column 1 shows high-field parcelation of the 11T atlas; column 2 shows BIOCARD population atlas deformed onto the 11T atlas transferring 11T high-field labels; column 3 shows high-field atlas labels transferred to BIOCARD template.

**Figure 4:**
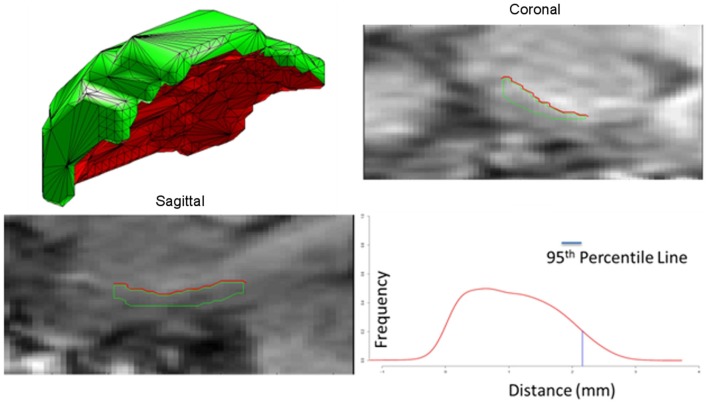
**Panel 1** shows an example of the surface meshes, generated from segmentations of the entorhinal cortices (ERC’s); **Panels 2 and 3** show the coronal and sagittal views of surface intersecting the MRI entorhinal cortex boundaries. Red lines depict the surface (red) lying on the gray-white matter interface. **Panel 4** shows the Labeled Cortical Distance Mapping (LCDM) from the gray matter segmentation and the surface, depicting the frequency of gray matter voxel as a function of distance to the gray-white surface. Thickness is calculated as the 95% point of the cumulative distribution function generated from the distance histogram.

**Figure 5:**
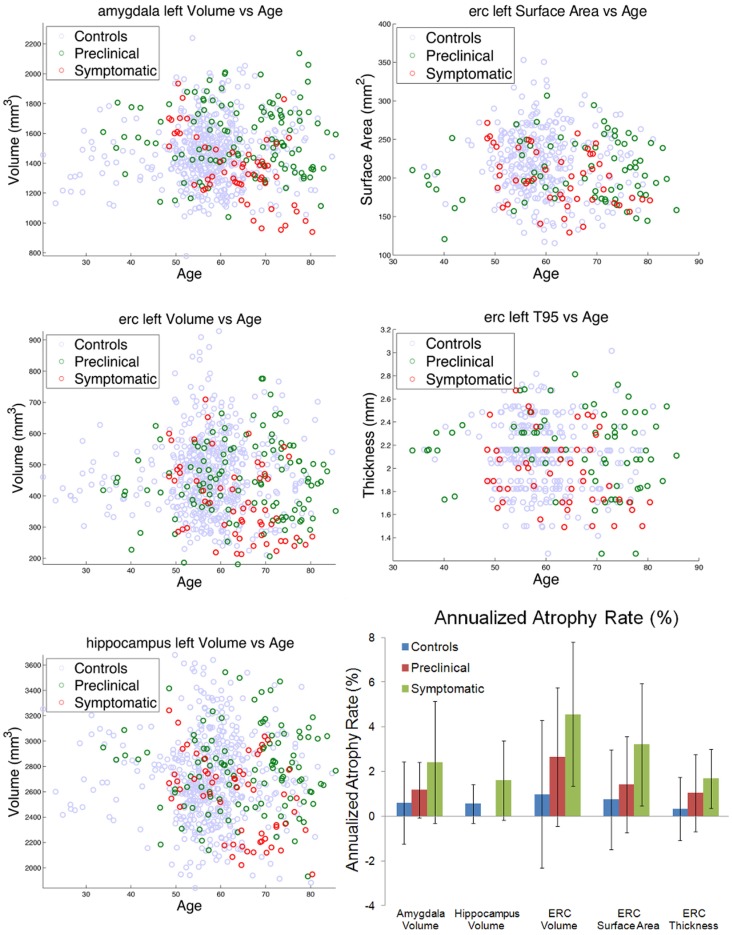
Left column shows amygdala (top), ERC (middle), and hippocampus (bottom), volumes for control (blue), preclinical (green), and symptomatic (red) subjects. Right column shows ERC surface area (top) and thickness derived from 95% of LCDM distances (middle). The bottom right panel shows atrophy rates for volumes in the amygdala, hippocampus, and ERC, and surface area and thickness in the ERC. Note the hippocampus volume for preclinical subjects was not significant and thus omitted.

The ERC is modeled as both a subvolume for morphometry as well as a thin laminar cortical structure with a laminar thickness dimension. Here, a closed smooth surface is generated from the gray matter volume from which the gray/white surface is extracted by curvature-based dynamic programing delineation of the extremal boundaries (Ratnanather et al., 2003) so that the surface closest to the white matter is retained. Figure 4 illustrates a reconstruction of the surface meshing of the ERC segmentation, which is cut to extract the surface (red) that lies on the gray–white matter interface. From this, we calculate the labeled cortical distance mapping (LCDM) (Miller et al., 2000, 2003) which calculates the set distances of the gray matters voxels to the cut surface. Several metrics for thickness can be extracted; the first is the constant thickness, zero-curvature approximation in which the thickness is approximated by the volume/surface-area, the second is the 95th percentile of the LCDM density profile. Figure 5 (right column) shows the surface areas (top row) and thicknesses (middle row) of ERC for control (blue), preclinical (green), and symptomatic (red) groups.

#### Mixed Linear Effects Models of Shape Morphometry

Diffeomorphometry and GPS provides geodesic coordinates, which are directly linked to the Jacobian of the mappings systems $X_{\begin{array}{c} \text{biocard}\\ \text{template} \end{array}}\overset{\varphi }{\underset{\varphi^{-1}}{⇄}}X_{\begin{array}{c} \text{biocard}\\ \text{population} \end{array}}$ defining the correspondences between coordinate systems in the population of control, preclinical, and symptomatic subjects. The Jacobian $\det\;\left(\frac{\partial \varphi }{\partial x}\right)$ measures the local expansion/compression of the target coordinate system relative to the template coordinate system, indexed over the surfaces of the three structures (the amygdala, ERC, and hippocampus) between the groups (control, preclinical AD, and symptomatic AD). The morphometry marker is modeled using several LME models (Miller et al., 2013; Younes et al., 2014a,b): (i) a longitudinal time-series model in which the time-series within each subject is modeled as a linear-slope as a function of age at the time of the scan and an intercept α + α′age for control, corrected by β + β′age for the disease category becoming a purely cross-sectional model when only a single scan for each subject is examined (or available) with testing between the groups determined by α, β alone, (ii) a changepoint model synchronizing all the population data at the time of clinical symptom onset and models the measures in the network of structures has having rate α + α′age up to changepoint time *t*_symptom_ − Δ and switching to α + (α′ + β′) age after changepoint. Δ is the unknown time in years before clinical symptom onset that the atrophy switches. For cross-sectional analyses, there is no age; for the longitudinal time-series then age is a descriptor; for changepoint detection an explicit variable is selected for the changepoint from one to the other model.

Each subject’s left and right structures is registered to the template, resulting in the computation of a normalized deformation marker *J_v_*(*s*) defined as the logarithm of the surface Jacobian indexed to the template surface at vertex *v* in subject *s*, measuring the logarithm of the local expansion/reduction around each vertex relative to the targets. The statistics on shape markers are constructed via linear, mixed effects modeling taking the mean of the atrophy as determined by an intercept and a rate. A single volume marker is also analyzed.

The mixed effects model (Bernal-Rusiel et al., 2012; Miller et al., 2013; Younes et al., 2014a,b) corresponds to representing the noise in measuring the shape marker as corresponding to two different processes: one associated to the time series within a subject and the second noise associated to the cross-sectional variation from subject to subject. The analysis includes age, gender, and log intracranial volumes as covariates, and computes statistics at each vertex of the triangulated template surface returning *p*-values corrected for multiple comparisons using permutation testing (Nichols and Hayasaka, 2003). The models are summarized as follows:

1. *Model I – longitudinal analysis*: Introducing group variables *g*(*s*) equal to 1 if subject *s* belonged to a diseased group, and to 0 otherwise, the LME model longitudinal model is given by:
  (3a) $$\begin{array}{l} J_{\mathrm{vj}}\left(s\right)=\left(\alpha_{v}+\alpha_{v}^{\prime }\text{,}a_{j}\left(s\right)\right)+\left(\beta_{v}+\beta_{v}^{\prime }\text{,}a_{j}\left(s\right)\right)g\left(s\right)+\;\gamma_{v}d\left(s\right)+\delta_{v}i\left(s\right)+\varepsilon_{\mathrm{vj}}\left(s\right), \end{array}$$
  where *a_j_* the age of the *j*th scan in the time series, with the covariates of gender *d*(*s*) and logarithm of intracranial volume *i*(*s*), and *v* is vertex marker number. The null hypothesis has β = β′ = 0 for all markers, while correcting for multiple comparisons.
2. The cross-sectional version has only the first scan (no time series) with no additional rate parameter α′ = β′ = 0, the model reducing to:
  (3b) $$J_{v}\left(s\right)=\alpha_{v}+\beta_{v}g\left(s\right)+\gamma_{v}d\left(s\right)+\delta_{v}i\left(s\right)+\varepsilon_{v}\left(s\right).$$
3. *Model II – longitudinal analysis with changepoint*: With changepoint, then the indicator (Heaviside) function (rather than group) determines regime change.
  (4) $$\begin{array}{llll} J_{\mathrm{vj}}\left(s\right) & =\alpha_{v}+\alpha_{v}^{\prime }\text{,}a_{j}\left(s\right)+\beta_{v}^{\prime }\text{,}\left(a_{j}\left(s\right)-\left(t_{\text{symptom}}-\Delta \right)\right)H\left(a_{j}\left(s\right)-\left(t_{\text{symptom}}-\Delta \right)\right)+\gamma_{v}d\left(s\right) & & \;\\ & \;+\delta_{v}\text{i}\left(s\right)+\varepsilon_{\mathrm{vj}}\left(s\right), \end{array}$$
  where the Heaviside function *H*(*x*) = 1 for *x* ≥ 0 and 0 otherwise, and *a* = *t*_symptom_ − Δ is the age before clinical symptom time exhibiting biomarker changepoint. Δ is the random variable representing the time in years before clinical symptom of slope change.

In the longitudinal case, the noise is modeled as ε*_vj_*(*s*) = η*_v_*(*s*) + ζ*_vj_*(*s*), where η*_v_*(*s*) is a “random effect” measuring between-subject variation, and ζ*_vj_*(*s*) measures within-subject variation; both processes are centered Gaussian, variance $\rho_{v}\sigma_{v}^{\text{2}}$ and $\sigma_{v}^{\text{2}}$, respectively. The within-subject variation does not appear in the cross-sectional model. The model parameters $\theta_{v}=\left(\alpha_{v}\text{,}\;\alpha_{v}^{\prime }\text{,}\;\beta_{v}\text{,}\;\beta_{v}^{\prime }\right)\text{,}\;\sigma_{v}^{\text{2}}\text{,}\;\rho_{v}$ are estimated using maximum-likelihood, with the estimation procedure derived in the appendix for all dimensions, v the hypotheses in the likelihood ratio test. Evaluating the log-likelihood, Eq. 5 below, at the MLEs of the parameters gives the log-likelihood determined by the mixed sums of squares as described in Appendix 2 in the Supplementary Materials. For Models I and II, we test for the null hypothesis with $H_{v}^{\text{0}}:\beta_{v}^{\prime }=\beta_{v}=0$ for all *v*, while correcting for multiple comparisons. For volume testing, the logarithm of the volume is used. For the changepoint onset model, Δ is estimated as well. For Model II changepoint there is only one offset so $\beta_{v}=0$ under all hypotheses. The *p*-values are computed using permutation sampling (Nichols and Hayasaka, 2003) running until 10% accuracy is reached with high probability. The joint test statistic is computed by taking the log-likelihood difference between the null hypothesis $H_{v}^{\text{0}}:\beta_{v}^{\prime }=\beta_{v}=0$ and the alternative general hypothesis $H_{v}^{\text{1}}:\left(\beta_{v}^{\prime }\text{, }\beta_{v}\right)\neq \left(0,0\right)$, computing

(5) $$S_{v}=L_{v}^{{\text{H}}_{\text{1}}}-L_{v}^{{\text{H}}_{\text{0}}}\text{.}$$

For the linear effects model, the log-likelihood difference between the hypotheses $H_{v}^{\text{1}}\text{:}\beta_{v}\neq 0$ and the null is equivalent to computing, for each coordinate *v*, the logarithm of ratio of the residual variance for the complete $H_{v}^{\text{1}}$ hypothesis to the one obtained from the null hypothesis. The *p*-values are computed by random permutation of the residuals, correcting for multiple comparisons. To compute *p*-values, S* = max_*v*_S_*v*_, the global statistic maximizing over shape coordinate *v*, is computed for a large number of permutations of the subjects randomizing the model residuals, with the *p*-value given by the fraction of times S* is larger than the value obtained; see section A1.4 in Appendix A in Younes et al. (2014a).

This permutation testing provides a conservative estimate on the set of vertices *v* on which the null hypothesis is not valid at a 5% family wise error rate (FWER). This set is defined by *D* = {*v*: *S_v_* ≥ q*} where q* is the 95th percentile of the observed value over the permutations (Nichols and Hayasaka, 2003).

For the cross-sectional Model I, with spatially normalized deformation marker *J_v_*(*s*) measuring the amount of expansion/atrophy at vertex *v* of the template surface in registering it to subject s, then the group variables *g*(*s*) = 1 if subject *s* belongs to the symptomatic group, and 0 otherwise belonging to the control group. The covariates are gender and intracranial volume as above with residual noise ε*_v_*(*s*) Gaussian distributed with variance $\sigma_{v}^{2}$, then

(6a) $$J_{v}\left(s\right)=\alpha_{v}+\beta_{v}g\left(s\right)+\gamma_{v}d\left(s\right)+\delta_{v}i\left(s\right)+\varepsilon_{v}\left(s\right)$$

Letting $\varepsilon_{\nu }^{\text{0}}\left(s\right)$ denoting the residuals for the model under $H_{v}^{0}:\beta_{v}=0$ permuting the residuals under *H*^0^ = null, with π a random permutation of the subjects gives

(6b) $$J_{v}^{\pi }\left(s\right)=\alpha_{v}^{\text{0}}+\gamma_{v}^{\text{0}}d\left(s\right)+\delta_{v}^{\text{0}}i\left(s\right)+\varepsilon_{v}^{\text{0}}\left(\pi_{s}\right)$$

We test for the null hypothesis with $H_{v}^{0}:\beta_{v}=0$ for all *v*. To compute *p*-values, S* is computed for a large number of permutations of the residuals, with the *p*-value given by the fraction of times S* is larger than the value obtained with the true groups.

## Results

### Volumes and atrophy rates

The left column of Figure 5 shows the volumes of amygdala (top row), ERC (middle row), and the hippocampus (bottom row) for the left temporal lobe structures. The control, preclinical, and symptomatic subjects are shown as blue, green red circles respectively. The top two rows of the right column show the surface areas and thickness of the ERC.

From these volumes, atrophy rates can be calculated since within-subject volumes in the time-series are available. For computing atrophy rates, only subjects with three or more scans were included. The bottom right panel of Figure 5 shows a summary of the atrophy rates for linear fits of the amygdala, hippocampus, and ERC volumes as a function of scan number for the control, the preclinical group, and the symptomatic group. Left column shows the total atrophy of amygdala in mm^3^ and percentage, respectively; right column shows the same for the hippocampus. Preclinical the atrophy rates in the amygdala are 0.6% in control, increasing to 1.2% and doubling again to 2.4%. The hippocampus atrophy rate in the symptomatic group has an atrophy rate of 1.6% which is more than double than that of the control group (0.7%). The rightmost part of the bottom panel of Figure 5 shows the summary of the bilateral results for the atrophy rates in percentages for the ERC, as volumes in mm^3^, surfaces in mm^2^ and thickness in mm. The ERC shows the most extreme atrophy increasing from 1% in control to 2.7% in the preclinical group to 4.6% in symptomatic group.

### Differential expression of degenerative change across structures

Clearly, the volume and atrophy rates signals the fact that degeneration is expressed differentially across the structures. In addition, there is sufficient measurement and anatomical variation in the markers that likelihood modeling to calculate the statistical significance of the apparent atrophy rates and atrophies associated to the disease states is required. So the LME models of I, II modeling the Jacobian and volume markers are used. For studying populations, the distribution of high-dimensional markers of diffeomorphometry presents significant challenges. While single volume numbers can be averaged across subjects, the morphometry markers must be synchronized across the population, requiring registration to the common template coordinates which is performed by rigidly aligning volumes and creating a single family of template shapes (Ma et al., 2010); see Figure 10 for sample template shapes. To examine the differential manifestation of neurodegeneration across the network of structures in the groups, the cross-sectional linear effect Model I is the simplest using only the first scan for each subject. Table 1 presents the *p*-values from cross-sectional testing (only first scan included) between the control and symptomatic groups based on the volume (column 2) and Vertex (column 3) morphometry measures. The columns list the *p*-values. For the cross-sectional Model I, left and right structures for each control and symptomatic subject were registered to the template giving spatially normalized deformation marker *J_v_*(s) measuring the amount of expansion/atrophy at vertex *v* of the template surface in registering it to subject *s*. Taking the group variables, *g*(*s*) = 1 if subject *s* belongs to the symptomatic group, and 0 otherwise belonging to the control group with the covariates as gender and intracranial volume as in Eqs 6a and 6b.

**Table 1:** ***p*-value of cross-section linear mixed-effects morphometry modeling including first scan only from Model I comparing control and symptomatic groups**.

Cross-section Model I (first scan only)	Volume	Vertex
Control versus symptomatic	*p*-Values	*p*-Values
Amygdala (L)	0.04	0.009
Amygdala (R)	0.08	0.003
Hippocampus (L)	0.06	0.21
Hippocampus (R)	0.1	0.0005
ERC (L)	0.00002	0.0002
ERC (R)	0.002	0.0005

*The table presents the *p*-values from cross-sectional testing of linear mixed-effects model for control versus symptomatic groups based on the volume (column 2) and vertex (column 3) morphometry measures. Columns list *p*-values for the volume markers (1 dimension per structure) and vertex (750–1500 dimensions per structure)*.

The *p*-values generated from permutation of the vertex based linear effects deformation markers of Eqs. 6a and 6b are shown in the rightmost columns of Table 2; the volume results in which *J* is replaced by the structure volume are shown in column 2. For volume statistics no multiple testing correction is required (1 volume dimension versus the 750–1500 vertex dimensions).

**Table 2:** **Differences in estimated onset of morphometric change in relationship to symptom onset for the amygdala, ERC, and hippocampus for Model II**.

Changepoint Model II	Volume		Vertex	
	*p*-value	***Δ*** ***±*** SD	*p*-value	***Δ*** ***±*** SD
Amygdala (L)	0.0005	6 ± 2.6	0.00005	4 ± 1.4
Amygdala (R)	0.007	3.5 ± 4.0	0.0024	3.5 ± 1.9
Hippocampus (L)	0.019	3 ± 2.4	0.035	3 ± 0.9
Hippocampus (R)	0.13	5.5 ± 3.8	0.029	5 ± 1.5
ERC(L)	0.000025	7 ± 3.7	0.000025	9 ± 1.6
ERC (R)	0.0056	8.5 ± 4.9	0.006	8 ± 3.2

*Onset is shown as average ± SD (Δ ± SD). Results based on groups with 221 control, 50 MCI, and 20 AD versus previous (230 control, 49 MCI, and 17 AD); based on bootstrap averages and rounding to integer*.

**Figure 6:**
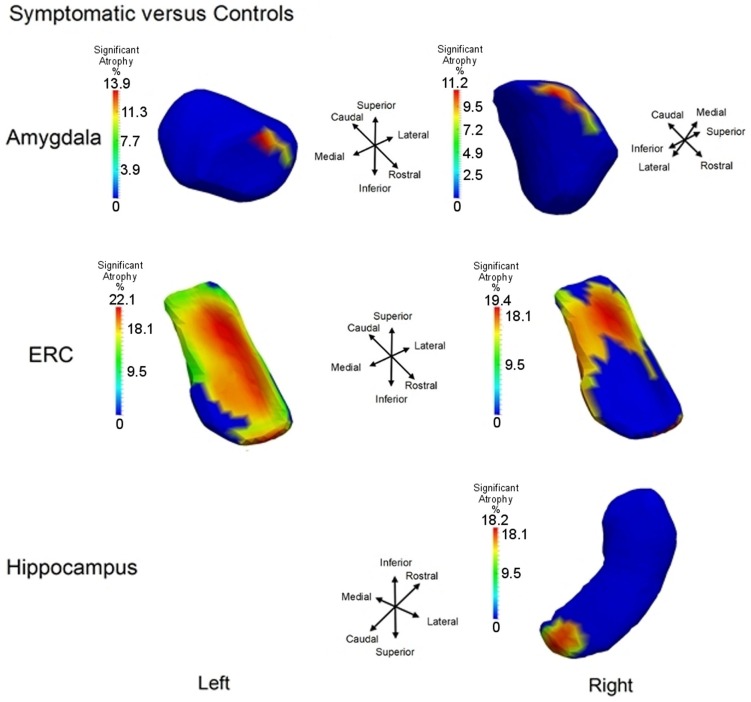
**Longitudinal Model I cross-section, first scan only, symptomatic versus control: Atrophy visualization FWER 5% as measured by testing group model testing ***α***_*v*_ versus ***α***_*v*_ + ***β***_*v*_ showing atrophy relative to control template of symptomatic as signaled by Jacobian of atrophy and atrophy rate demonstrating percentage decrease of control to symptomatic group**. Top row shows amygdala, middle row entorhinal cortex, and bottom row hippocampus. Only right hippocampus is shown because the other side is not significant.

To illustrate the differential spatial extent of significant atrophy, Figure 6 shows the vertices satisfying the FWER criterion at 5% (this means that, with probability 0.95, there is no false detection among the vertexes considered as significant); also shown are the vertex markers that were significant with the color of the vertices given by the atrophy measure −β*_v_*. Since the left hippocampus was marginally significant, we deleted the FWER plot (bottom left panel). In almost all cases, the sensitivity of the vertex based method is illustrated by the rejection of the null hypothesis. The sensitivity of the ERC compared to the hippocampus for example is demonstrated by its extreme significance. Also, notice that all change is represented via monotonically decreasing relative volume of groups relative to control corresponding to the warm red colors (blue denotes no change, red shrinkage), with the ERC demonstrating as much as 18% decrease in local surface area vertex by vertex relative to the control group. The amygdala shows less change, maximally 12%.

### The “when” of network degeneration via temporal changepoint modeling

The monotonic decrease in relative volume of the disease group to the control group as indicated by the maximal surface area decrease of 18% locally is what we term the atrophy. It acts as our localized marker or signal associated to the underlying neurodegeneration process. The localized markers shown in Figure 6 are all derived from one sample per subject. There is no use of the fact that many subjects, at least 80 have three scans or more corresponding to a time-series from which a temporal ordering of change can be estimated. Our changepoint model attempts to do this by introducing a global time ordering to the change which must be estimated and using the cross-sectional responses of each individual to fill in the information to this temporal ordering.

Figure 5 illustrates the massive confounder that an individual’s age presents in understanding the neurodegeneration process as manifest by the disease. While age is a risk factor for disease, the profile of neurodegeneration is individualized, alignment of atrophy measures cross-sectionally has an associated inherent confounding variation appearing to mask the trend. The temporal ordering changepoint model we have developed attempts to synchronize the change dependence of individuals according to their clinical time rather than their ages. So, we model the processes of atrophy in reverse, proceeding backwards or in negative time relative to the synchronizing clinical time, thereby allowing us to align all of the subjects based on their estimated clinical time. This acts to remove the confounder associated to the fact that different subjects follow their own time courses determined by when their clinical symptom time is.

The changepoint Model II assumes that there is a regime change in the neurodegeneration process that occurs at some random time for all subjects in the disease populations, an unknown Δ years before clinical symptom time. This random time Δ preceeding symptom time is subject dependent as well as – structure in the network – dependent. It must be estimated. The changepoint models neurodegeneration so that for ages age < *t*_symptom_ − Δ, the atrophy process follows one regimen or slope given by $\alpha_{v}+\alpha_{v}^{\prime }\text{age}$, and for individuals after their changepoint time age ≥ *t*_symptom_ − Δ they follow a separate regimen with the atrophy slope increasing with identical intercept $\alpha_{v}+\left(\alpha_{v}^{\prime }+\beta_{v}^{\prime }\right)$ age. We can view the symptomatic time as the *X* = 0 ordinate or origin for all subjects, with time progression preceding clinical symptom time expressed relative to the clinical symptom time or origin. So, *X* = 0 clinical symptom time plays the role of the synchronizing event for the population. The slope parameters $\alpha_{v}^{\prime }\text{age}$ before changepoint are estimated from the control subjects and from preclinical and symptomatic subjects before changepoint Δ and the slope parameters $\left(\alpha_{v}^{\prime }\text{+}\beta_{v}^{\prime }\right)$ age after onset are estimated from the preclinical and symptomatic populations after changepoint, the changepoint time Δ being determined so that the data likelihood is maximized. The changepoint model was applied to all of the structures with permutation testing calculated for rejection of the null hypothesis with $H_{\text{v}}^{0}:\beta_{\text{v}}=0$ for all *v*. Table 2 shows the result of applying Model II for changepoint estimation, with the associated *p*-values depicted for each structure in columns 2 and 4, corrected for multiple hypotheses. The onset time and SDs were estimated for each changepoint model Δ ± SD and are in columns 3 and 5. We see in all cases, the likelihood ratio testing based on the high dimensional vertex markers is strongly significant for ERC and amygdala, with the hippocampus giving the weakest rejection of the null hypothesis. Not only is the ERC the most sensitive signaler of significant atrophy rate change during progression, but also earliest as indicated by the time before clinical symptom onset being on the order of 8 years. The left ERC shows the most significant changepoint profile, occurring earliest.

**Figure 7:**
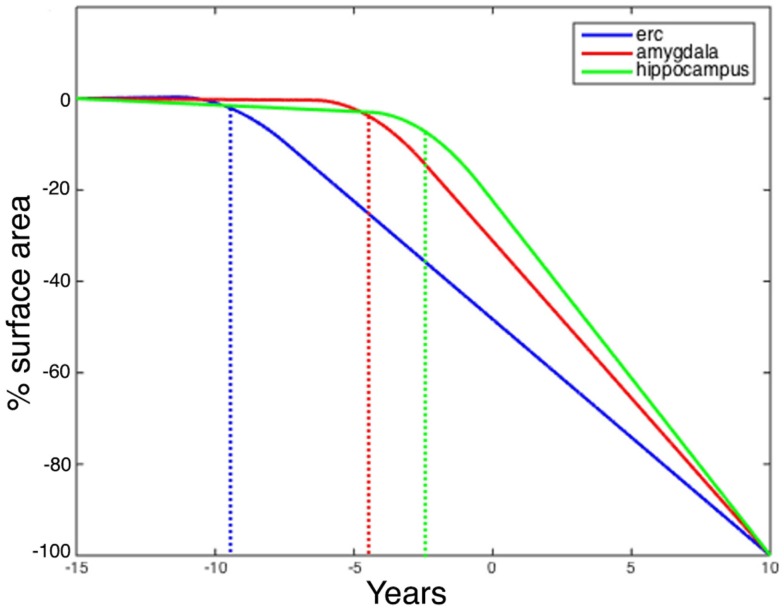
**Longitudinal changepoint Model II fits of left amygdala (red), ERC (blue), and hippocampus (green)**. The Y-axis shows percent decrease of surface area relative to control averaged over template vertices Morphometry markers plotted using subject’s changepoint time Δ relative to clinical onset time *X* = 0 occuring at the sharp bend). Changepoint occurs at the sharp bend in curves between Δ:8 and 9 years for the ERC. The median changepoint times (before onset) in the bootstrap samples were ERC: 9 years (left) and 10 years (right), Amygdala: 4.0 years (left) and 3 years (right), Hippocampus: 2.5 years (left) and 5.0 years (right). Graphs have been scaled so that they all start at 0% decrease and end at 100%.

**Figure 8:**
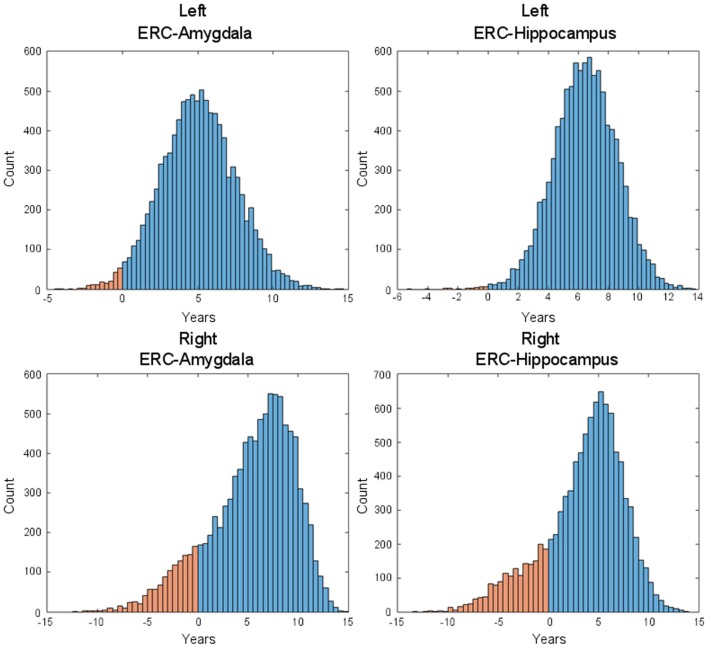
**Histogram of time-difference between estimated changepoints for left-side (first row) and right-side (second row) structures (left: ERC – Amygdala; right: ERC – Hippocampus) based on 10,000 bootstrap samples**. On the left side, onset times were found larger in ERC than in Amygdala 98.1% of the time (87.5% on the right side) and larger in ERC than in hippocampus 99.7% of the time (82.5% on the right side), with respective median difference of 5.0 and 6.5 years (6.2 and 4.3 on the right side).

Figure 7 shows the changepoint model estimated from the population of left temporal lobe structures, ERC (blue), amygdala (green), and hippocampus (red). The models shown are based on the estimated parameters $\alpha_{v}\text{,}\;\alpha_{v}^{\prime }\text{,}\;\beta_{v}^{\prime }\text{,}\;\Delta$ whose *p*-values are given in Table 2, but have been scaled to show 100% change for all three curves. The model curves are depicted for each structure with slope parameters before changepoint and after changepoint, each with their own changepoint time Δ relative to synchronizing clinical symptom time denoted as *X* = 0 on the *X*-axis. The *Y*-axis shows surface area percent decrease averaged over the vertices relative to the original template. Changepoint occurs at the sharp bend in curves between Δ: 9 and 10 years for the ERC, with a SD of 1.5–3.5 years (see Table 2). The left ERC appears to have a strong atrophy rate after change point. The median changepoint times (before onset) in the bootstrap samples were 9 years (left) and 10 years (right) for ERC, 4.0 years (left) and 3 years (right) for amygdala, and 2.5 years (left) and 5.0 years (right) for hippocampus. Figure 8 shows a histogram of time-differences between estimated changepoints for the ERC compared to amygdala (left column) and ERC compared to hippocampus (right column) for 10,000 bootstrap samples for left (top) and right (bottom) sides. For the left, onset times were larger in ERC than in amygdala 98.1% of the time and larger in ERC than in hippocampus 99.7% of the time with a median difference of 5.0 and 6.5 years. On the right, it was 87.5% and 82.5% for amygdala and hippocampus, respectively, with a median difference of 6.2 and 4.3 years.

**Figure 9:**
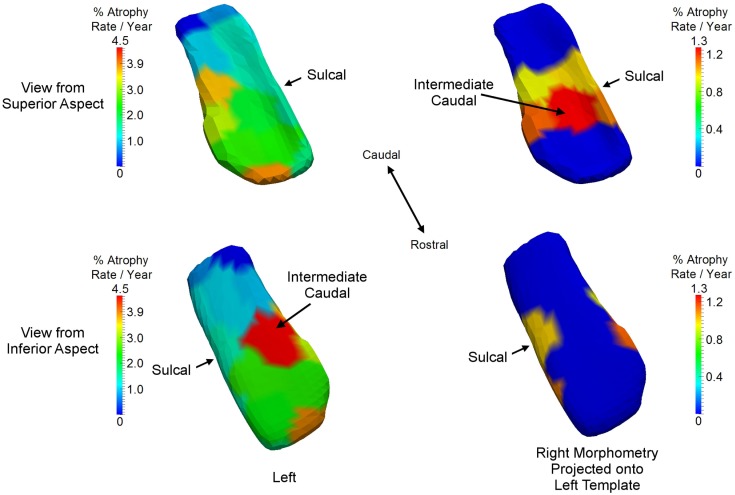
**Longitudinal changepoint Model II depicting atrophy rate with atrophy rate annualized per year of left ERC on left ERC template (left column) and right ERC projected on left ERC template (right column) on the statistically significant vertices based on the FWER of 5% testing of vertices**. Non-significant vertices are depiced as blue. Significant vertices are colored with the annualized atrophy rate $\beta_{v}^{\prime }$ occurring after changepoint. Top and bottom rows, respectively, shows superior and inferior views. Left ERC has maximum value of $\beta_{v}^{\prime }=\text{4}.\text{5}\%$ atrophy rate per year; right has maximum value of $\beta_{v}^{\prime }=\text{1}.\text{3}\%$ atrophy rate per year. Sulcal and intermediate caudal are two partitions in high-field atlas.

The ERC is clearly differentially more sensitive than the other structures in the network in that atrophy occurs earliest, but we also see that the left ERC appears to be more strongly significant in *p*-value when tested against the null hypothesis with $H_{v}^{\text{0}}:\beta_{v}^{\prime }=0$ for all *v*. The atrophy rate slope of the left ERC across the vertices is large. Figure 9 depicts the annualized atrophy rates of the ERC vertices $\beta_{v}^{\prime }$, which are most significant under the FWER at 5% permutation testing of the changepoint model. The significant vertices contributing to the strong *p*-values under the FWER at 5% criterion are colored with the annualized atrophy rate $\beta_{v}^{\prime }$ occurring after changepoint; non-significant vertices are depicted as blue. The top and bottom rows of Figure 9, respectively, show the superior and inferior views. Notice that the left ERC has a maximum value of $\beta_{v}^{\prime }=\text{4}.\text{5}\%$ atrophy rate per year as compared to the right having a maximum value of $\beta_{v}^{\prime }\text{=1}.\text{3}\%$ atrophy rate per year. It appears as if the changepoint model for neurodegeneration in the ERC is being most strongly signaled by the annualized atrophy rate parameter.

### The “where” of neurodegeneration via high-field atlasing

**Figure 10:**
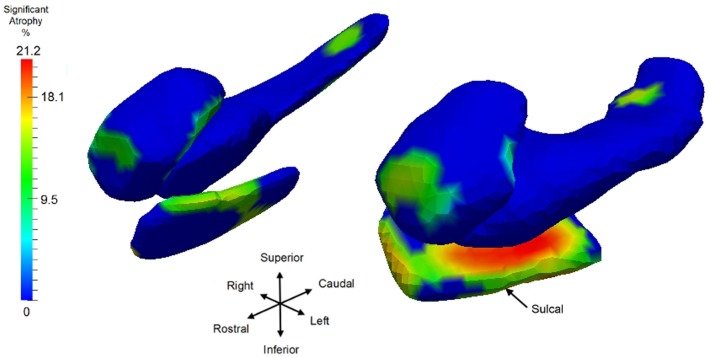
**Longitudinal time-series Model I, symptomatic versus control with a view of the significant vertices as measured by log-Jacobian change between control and symptomatic group**. Significant vertices showing consistent local volume decreases as determined by the FWER at 5% linear effects Model I; blue vertices represent vertices which are not significant. Color codes percentage decrease of surface area from control to symptomatic, with color given by $-\left(\beta \text{+}\beta_{v}^{\prime }\;\bar{\text{age}}\right)$, with $\bar{\text{age}}$ the average age of the symptomatic group. Structures placed into coordinates of one brain depicting the relative relationships of the most significant changes in color from blue to red. Maximum changes of relative surface area of symptomatic population to control population are 21% for ERC; the maximum for hippocampus is 15.5%, and 15% for amygdala; all significant change (non-blue) is atrophy. Sulcal region denoting the most lateral portion of ERC is depicted.

Having examined the *when* of the neurodegeneration of the network of structures, we now examine the *where* in the network the localized atrophy is occurring. Returning to the longitudinal time-series, we use Model I to define the single most sensitive markers for signaling where change is occurring, independent of the temporal order onset model. Notice Model I does not include the extra parameters of a changepoint or regime time within the longitudinal time-series of each individual. The vertex deformation marker *J_v_*(*s*) is modeled as $\alpha_{v}\text{+}\alpha_{v}^{\prime }\left(s\right)a\left(s\right)+\beta^{\prime }\left(v\right)a\left(s\right)g\left(s\right)$, with the group category of symptomatic determining the extra slope rate of annualized atrophy. In this model we perform the permutation testing for computing *p*-value on the mixed effects model by testing against the null hypothesis with $H_{v}^{\text{0}}:\beta_{v}^{\prime }=0$ for all *v* and permuting the residuals checking for the number of times the true labeling under the model is rejected, correcting for multiple comparisons. Figure 10 shows atrophy visualization of the FWER significant vertices for symptomatic and control groups. For the statistically significant vertices as defined by the FWER at 5%, the vertices are colored with the atrophy measure with value $-\left(\beta \text{ +}\beta_{v}^{\prime }\;\bar{\text{age}}\right)$ where $\bar{\text{age}}$ is the average age in the symptomatic AD population, with the natural log of Jacobian of atrophy rate at that vertex plotted as a percentage decrease of the control to symptomatic group. The values $\beta \text{,}\;\;\beta_{v}^{\prime }$ are estimated according to the Model I using maximum-likelihood estimation (see Appendix 2) with a parameter value at each vertex. In Figure 10, the structures are placed into the coordinates of one brain depicting the relative relationships of the most significant changes shown in color from blue to red, with blue depicting the non-significant vertices as measured by the FWER at 5% procedure. Maximum change of relative surface area of symptomatic population to control population is 21% in the ERC, with 15% maximal changes in the hippocampus and amygdala. All vertices for which change is not significant in the permutation testing are denoted as blue; change is one direction, atrophy.

Table 3 shows the *p*-value significance for substructures between the control versus the symptomatic group category based on the longitudinal time-series Model I for all scans modeling the log Jacobian of change as $\alpha_{v}\text{+}\alpha_{v}^{\prime }a_{j}\left(s\right)+\beta_{v}^{\prime }a_{j}\left(s\right)g\left(s\right)$. The permutations on residuals are tested under the null hypothesis with $H_{v}^{\text{0}}:\beta_{v}^{\prime }=0$ for all *v*, correcting for multiple comparisons. Significance is shown in column 2 for volume only (one dimension per structure) and in column 3 for the vertex-based modeling (750–1500 dimensions per structure). All structures are significant with the significance increasing from the volume testing to the vertex based high-dimensional markers.

**Table 3:** **Morphometry measures comparing normal group versus symptomatic group via linear mixed-effects longitudinal time-series Model I**.

Longitudinal time-series Model I *p*-values	Volume	Vertex
Control versus symptomatic group	
Amygdala (L)	0.005	<0.00001
Amygdala (R)	0.0020	0.00018
Hippocampus (L)	0.055	0.019
Hippocampus (R)	0.1330	0.00103
ERC (L)	0.00008	<0.00001
ERC (R)	0.0060	0.005

The table presents the *p*-values from LME longitudinal time-series Model I testing control versus symptomatic group based on the volume (column 2, 1 dimension) and vertex (column 3, 750–1500 dimensions) morphometry markers.

### Comparing to braak and braak staging via high-field atlasing

**Figure 11:**
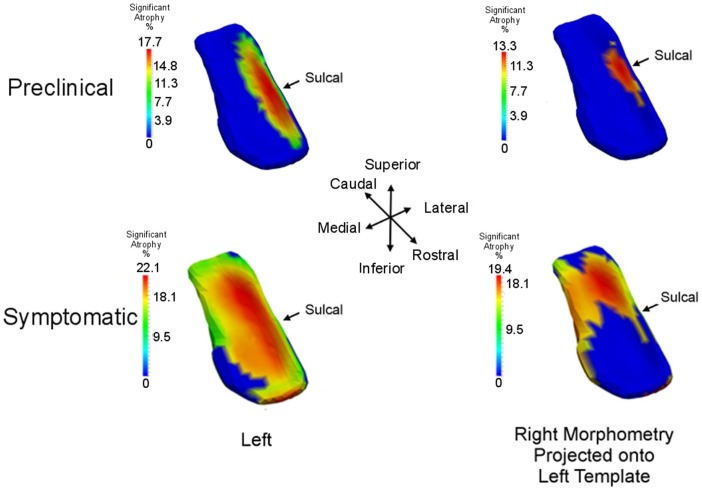
**Longitudinal time-series Model I: spreading of significant morphometry marker from preclinical (top row) to symptomatic (bottom row); left column shows left ERC population change in left ERC template, right column shows right ERC projected onto the left ERC template**. Atrophy visualization for FWER at 5% as measured by linear effects. Color codes percentage decrease of surface area from control to preclinical group (top row) and control to symptomatic group (bottom row), given by $-\left(\beta \text{+}\beta_{v}^{\prime }\;\bar{\text{age}}\right)$, $\bar{\text{age}}$ the average age of the preclinical group (top) and symptomatic group (bottom). All significant change (non-blue) is atrophy with maximum changes of 18% for preclinical and 22% for symptomatic; blue is non-significant vertices with all change atrophy. Sulcal partition is S from high-field atlas.

**Figure 12:**
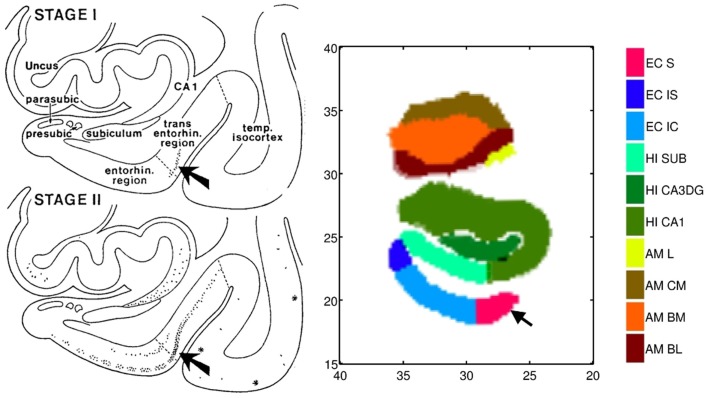
**Left**: Braak and Braak staging of AD neuropathology based on the pattern of neurofibrillary change adapted from Figure 9 of Braak and Braak (1991) who suggested that stages I–II characterize the silent periods of AD with stages III–IV corresponding to preclinical AD and stages V–VI for fully developed AD. Note that the progression is from lateral ERC and spreads medially. **Right**: Section through the high-field atlas of Figure 2 at position 17 mm along the axis. Purple red shows the S sulcal region in the high-field atlas which in the preclinical phase is the only region exhibiting significant morphometry marker change in range of 15% atrophy (top row, Figure 11). Notice the placement of the amygdala in the high-field atlas with basolateral, basomedial, and centromedial subfields.

We use high-field 11T atlasing to demonstrate that the network changes are occurring at the lateral most extent of the medial temporal lobe network in the high field atlas. Specifically, we use the high-field parcelation of the ERC that we have developed based on eight subfields derived: prorhinal, lateral, intermediate, sulcal, and medial – Pr, L, I, M, and S with subareas within these subfields demarcated. Figure 11 shows the ERC in the same orientation as the ERC in the top row of Figure 9, depicting the transition from preclinical (top row) to symptomatic (bottom row). The left and right columns show the left and right ERC population FWER 5% from the BIOCARD population, with the right ERC projected onto the left template so it appears in similar left orientation. Color intensity shown on the templates are proportional to atrophy level, given by $-\left(\beta +\beta_{v}^{\prime }\;\bar{\text{age}}\right)$, where $\bar{\text{age}}$ is the average age in the study and are estimated according to the cross-sectional Model I using maximum-likelihood estimation (see Appendix 2) with a parameter value at each vertex. These values are all positive indicating that group difference reflects atrophy. The left ERC is more significant as demonstrated by the atrophy range percentage, but also demonstrates the strong spread from the preclinical case in the lateral part of the ERC (top row) to a greater extent of the ERC (bottom row). The maximum value of change is 17% for preclinical and increases to 22% at the maximum value for the symptomatic case. The maximum value for the preclinical occurs at the sulcal region which is denoted by the arrow at the most lateral extent. The panels show only the vertices on the surfaces of the templates which are statistically significant for atrophy as measured by the FWER at 5% permutation testing. Non-significant vertices have zero intensity and are shown in blue. The strongly significant change in the preclinical group in the sulcal region of the high-field partition (indicated by S red color in Figure 3) is in the lateral extent of the ERC. This spread extends medially in the transition from preclinical to symptomatic. This is consistent with the Braak and Braak (1991) staging of the preclinical case as illustrated in Figure 12. The left panel of Figure 12 shows stages 1 and 2 of the cellular changes in the earliest preclinical phase (indicated by the arrow) from Braak and Braak. Stage 1 (top) is similar to the earliest periods of our imaging measurements. Here the changes are in the layer II of the lateral part of the ERC and trans-ERC. This then spreads from lateral to medial in the ERC in Stage 2. The right panel shows a planar section through the high field atlas (see Figure 2) depicting the location in the atlas of the EC S (red), IC (cyan), IS (blue) regions. The arrow is placed at the lateral most extent of the sulcal S region and depicts the location that the 5% statistically significant FWER diffeomorphometry population marker is signaling (as depicted in top row of Figure 11).

## Discussion

An important question in the field of neurodegeneration is the pattern of regional degeneration in the earliest phases of disease. The notion of cell–cell interaction hypothesis is being examined in several neurodegenerative illnesses, where the pathogenesis depends on brain circuitry (Polymenidou and Cleveland, 2011) in a prion- like manner [see Brundin et al. (2010) for a general review]. In this model, pathogenesis would depend, at least in part, on brain circuitry. Determining whether neurodegeneration in AD follows a consistent circuit-based pathway is important for identifying outcomes that might be measured during an intervention trial which directly targets the brain. Our findings seem to support this notion of circuit based locality of the spread. The fact that the ERC as a structure is most sensitive in terms of localized change associated to disease progression across the spectrum of AD, and earliest structure to be changing is manifests in the largest atrophy rates in percentages in the symptomatic group and the <0.00001 *p*-values for the high-dimensional surface-based vertex measures signaling localized atrophy in the symptomatic group. Based on onset analysis, atrophy rates and *p*-value significance for the hippocampus and amygdala demonstrate morphometric change later in the disease progression.

With respect to the ERC (Small et al., 2011), it is worth noting that layer II connects to the dentate gyrus (DG) in the hippocampal formation via the perforant pathway, and in turn the DG connects to the CA3 whose neurons connect with other CA3 neurons along the hippocampal axis or CA1, and finally CA1 connects to the subiculum. Layer II also connects with CA3 while layer III projects to the CA1 and subiculum. The ERC also receives inputs from the amygdala in an anterior–posterior gradient, which allows it to connect with the hippocampus. The subfield parcelation of the ERC in our high field atlas actually reflects the differences in the cellular and neuronal densities in Layer II based on stereological analysis. For example, in the Pr subfield, Layer II is thin and takes up 5% of the subfield volume while in the L subfield, it is larger in size relative to the I subfield; Ir takes up 10% of the volume and Is neurons are small; Mr and Mc it becomes thinner and more continuous; and finally in S the neurons are relatively medium sized. So, a localized shape difference in the ERC may be a manifestation of the changes in Layer II of the ERC subfields as has been suggested by several studies including Braak and Braak (1991). However, additional studies that correlate shape diffeomorphometry results with histopathological analysis of the same dataset similar to that done for Area 46 of in a fetal irradiation macaque model (Selemon et al., 2013) will be necessary.

These MRI findings are consistent with the patterns of pathology with brain regions demonstrating heavy deposits of neurofibrillary tangles (Arnold et al., 1991; Braak and Braak, 1991; Price and Morris, 1999). Our high-field localized shape morphometry demonstrated in the ERC are consistent with histological findings indicating that AD begins to manifest itself in the ERC (Gomez-Isla et al., 1996). The transentorhinal region referred to by Braak and Braak (1991) connects to the lateral boundary that shows significant atrophy in these analyses. Associating the high-field atlas with the results in Figures 8, 10, and 11 demonstrate this clearly. The vertex-based diffeomorphometry allows us to see the localized atrophy giving rise to the statistically significant visualization of the regions in the hippocampus and amygdala which are demonstrating change. This includes the CA1 and subiculum of the hippocampal region and its interface to the basolateral and basomedial regions of the amygdala. It is interesting to note that this rostral end region of the hippocampus actually abuts the Ic subfield of the ERC, which we observed to be significantly affected (Figure 9). Also shape changes, specifically reduced cortical thickness, has been observed in the transentorhinal cortex which is more lateral to the focus of this study but in the later Braak and Braak stages in subjects with MCI (Yushkevich et al., 2015). This provides strong support that shape diffeomorphometry modeling can be a powerful way of demonstrating the cell–cell circuitry model of AD. We have previously demonstrated via amygdala 7T and 11T atlases that preclinical change in amygdala was occurring in the basolateral and lateral areas (Miller et al., 2014a). We have demonstrated that the amygdalar atrophy changes tend to occur between the junctures of the networks or structures.

These findings are consistent with the cell–cell hypothesis, which has been advocated as a model of the spread of neurodegeneration in networked deep brain structures in Huntington’s Disease (Ross et al., 2014), Parkinson’s Disease (Visanji et al., 2013; Kordower, 2014), AD (Yin et al., 2014), and depression (Small et al., 2011). It is believed that the spread via intercellular communication in which plaque transmission from cell to cell occurs in a prion-like manner (Small et al., 2011). At the same time, excitatory and/or inhibitory processes may occur in a similar manner. However, further development is needed in order to better visualize the laminar structure within this network to determine the selectivity of involvement in early disease and the pattern of change over time. If neuroimaging studies such as the BIOCARD study or PREDICT-HD (Younes et al., 2014b) can pinpoint the initial substrates of the disease, it may be possible to develop therapeutic interventional strategies to minimize the spread of disease from these substrates.

## Conflict of Interest Statement

Susumu Mori and Michael I. Miller own “AnatomyWorks.” Susumu Mori is its CEO. This arrangement is being managed by the Johns Hopkins University in accordance with its conflict of interest policies. Other authors declare that the research was conducted in the absence of any commercial or financial relationships that could be construed as a potential conflict of interest.

## Supplementary Material

The Supplementary Material for this article can be found online at http://journal.frontiersin.org/article/10.3389/fbioe.2015.00054/abstract
