# Supplementary material for: Network Neurodegeneration in Alzheimer’s Disease via MRI Based Shape Diffeomorphometry and High-Field Atlasing
Source: Front Bioeng Biotechnol. 2015 May 15;3:54. doi: 10.3389/fbioe.2015.00054 (PMC4515983; doi:10.3389/fbioe.2015.00054)
Supplement: Supplementary file 1 [file datasheet_1.pdf]

Network Neurodegeneration in Alzheimer's Disease via MRI based Shape Diffeomorphometry and High-Field Atlasing

Michael I. Miller*, J. Tilak Ratnanather, Daniel J. Tward, Timothy Brown, David S. Lee, Michael Ketcha, Kanami Mori, Mei-Cheng Wang, Susumu Mori, Marilyn Albert and Laurent Younes, the BIOCARD Research Team

*** Correspondence:** Michael Miller, Center for Imaging Science, Johns Hopkins University, 3400 N. Charles St. Clark Hall 301, Baltimore, MD, 21218, USA.
mim@cis.jhu.edu

1. Supplementary Material

Appendix 1: Computing the distance between surfaces and the algorithm for surface matching

The N error terms compute the mismatch between surfaces by assuming the deformed template and target surfaces have local parameterizations $S = q(u), u \in U,\ S' = q'(u), u \in U,$ with the distance between smooth coordinates based on the normals of the surfaces given by $N(u) = \partial_{u_1} q(u) \times \partial_{u_2} q(u),\ N'(u) = \partial_{u_1} q'(u) \times \partial_{u_2} q'(u), u = (u_1, u_2) \in U,$ with $\times$ the cross-product. The disparity between surfaces or normed error between S, S' is given by

$$E(S, S') = \iint_{UU} W(u, u') \langle N(u), N'(u') \rangle du du' \tag{1}$$

where W is the smoothing window over which the integral is computed, and $\langle \cdot, \cdot \rangle$ is the inner-product between normal vectors.

Since the vector space $v \in V$ of vector fields is spatially smooth it has a reproducing kernel defined as K implying that the variational minimizers of Eqn. (1) will involve the kernel (see below). The variational problem of Equation 1 is solved by representing the deforming surfaces as a dynamical system, with state $q_0, u \in U,\ q_t = \varphi_t(S_{temp}),\ q_0 = S_{temp}$. Denoting the 3×3 Jacobian matrix as $(Dv) = (\partial_{x_i} v_j)$, $(Dv)^t$ matrix transpose, the solution satisfies $t \in [0,1]$,

$$\begin{aligned} \dot{q}_t &= v_t(q_t) = \int_U K\big(q_t, q_t(u)\big) p_t(u) du, \\ \dot{p} &= -(Dv_t)^t \circ q_t p_t, \\ &\text{subject to } p_{t_i} = \nabla_q E\big(q_{t_i}, S^i\big), i = 1, \ldots N \end{aligned} \tag{2}$$

with $\nabla_q E\big(q_{t_i}, S^i\big)$ denoting the 3×1 gradient of the matching cost with respect to the state. The target surfaces enter through boundary conditions involving the state transforming the template.

Appendix 2: Linear mixed-effects Modelling for Group Comparisons: Control versus Preclinical and Control versus Symptomatic

<u>Calculation of the MLE parameters for the linear mixed-effects model:</u> The parameters $\alpha_v, \alpha'_v, \beta_v, \beta'_v, \sigma_v^2$ are estimated by maximum likelihood for all dimensions v for each of the two

hypotheses. Evaluating the log-likelihood in each case at the MLEs of the parameters gives log-likelihood essentially determined by the mixed sums of squares.

We describe the maximum likelihood estimation algorithm, focusing on the general hypothesis H_v^1. The null hypothesis H_v^0 is handled the same way, without the parameters β_v, β_v', or $\beta_v = \beta_v' = 0$. We also work with fixed v, since the models across shape coordinates do not share any parameter and can be estimated independently from each other. The model parameters for H_v^1 are then $\theta_v = (\alpha_v, \alpha_v', \beta_v, \beta_v'), \sigma_v^2$ and ρ_v; for H_v^0 then $\theta_v = (\alpha_v, \alpha_v', 0, 0), \sigma_v^2$.

We now describe the estimation procedure.

Let n denote the number of subjects, N_s the number of scans for subject s and N the total number of scans (the sum of all N_s). Let d be the number of variables in the linear model (d = 4 in our case) and K the dimension of the shape marker. Denote $Y_v(s)$ the vector with coordinates $y_{vj}(s)$ for $j = 1, \dots, N_s$ and define $\varepsilon_v(s)$. Rewrite the linear model for coordinate v as $Y_v(s) = X(s)\theta_v + \varepsilon_v(s)$, where $X(s)$ is an N_s by d matrix and θ_v a d-dimensional vector.

Let $\mathbf{1}_s$ denote the N_s-dimensional vector with all coordinates equal to 1. The covariance matrix of $\varepsilon_v(s)$ is the $N_s \times N_s$ matrix $A_v(s) = \sigma_v^2(Id + \rho_v \mathbf{1}_s \mathbf{1}_s^T)$.

One has $A_v(s)^{-1} = \sigma^{-2}\left(Id - \frac{\rho_v}{1+N_s\rho_v}\mathbf{1}_s\mathbf{1}_s^T\right)$ and $\det(A_v(s)) = \sigma_v^{2N_s}(1 + N_s\rho_v)$.

This implies that the log-likelihood of the sample is (up to an additive constant)

$$
\begin{aligned}
l = &-\sum_{v=1}^{K}\sum_{s=1}^{n}\frac{1}{2\sigma_v^2}\|Y_v(s) - X(s)\theta_v\|^2 \\
&+\sum_{v=1}^{K}\sum_{s=1}^{n}\frac{\rho_v}{2(1+N_s\rho_v)\sigma_v^2}\left(\mathbf{1}_s^T\left(Y_{v(s)} - X(s)\theta_v\right)\right)^2 \\
&-\frac{N}{2}\sum_{v=1}^{K}\log\sigma_v^2 \; -\frac{1}{2}\sum_{v=1}^{K}\sum_{s=1}^{n}\log\left(1 + N_s\rho_v\right)
\end{aligned}
$$

The procedure loops over the following two steps until convergence (which usually requires a small number of iterations)

Step 1: Least square estimation, updating all parameters except ρ_v.

This minimizes the likelihood with respect to θ_v and σ_v^2. Define the covariance matrices $S_{XX} = \sum_{s=1}^n X(s)^T X(s)$, $S_{XY}^v = \sum_{s=1}^n X(s)^T Y(s)$. Define also the row vector $\bar{X}(s) = \mathbf{1}_s^T X(s) = \sum_{j=1}^{N_s} X_j(s)$ where $X_j(s)$ is the jth row of $X(s)$, and the scalar $\bar{y}_v(s) = \mathbf{1}_s^T Y_v(s) = \sum_{j=1}^{N_s} y_{vj}(s)$.

Set $\bar{S}_{XX}^v = \rho_v \sum_{s=1}^n \bar{X}(s)^T \bar{X}(s)/(1 + N_s\rho_v)$, $\bar{S}_{XY}^v = \rho_v \sum_{s=1}^n \bar{X}(s)^T \bar{y}_v(s)/(1 + N_s\rho_v)$

Then, a direct computation shows that the least square estimator of θ_v is given by

$$\hat{\theta}_v = (S_{XX} - \bar{S}_{XX}^v)^{-1}(S_{XY}^v - \bar{S}_{XY}^v).$$

To estimate the variance, define the residual $R_v(s) = Y_v(s) = X(s)\hat{\theta}_v$. For a given v, let $\bar{R}_{v(s)} =$

$\mathbf{1}_s^T R_v(s) = \sum_{j=1}^{N_s} R_{vj}(s)$, then, one must take

$$\hat{\sigma}_v^2 = \frac{1}{N}\sum_{s=1}^{n}\|R_v(s)\|^2 - \frac{\rho_v}{N}\sum_{s=1}^{n}\frac{\bar{R}_s(s)^2}{1+\rho N_s}$$

Step 2: Update ρ_v with all other parameters fixed. Focusing on the part of the likelihood that depends on ρ_v, we see that $\hat{\rho}_v$ minimizes the function

$$\rho - \frac{\rho}{\hat{\sigma}_v^2}\sum_{s=1}^{n}\frac{\bar{R}_s(s)^2}{1+\rho N_s} + \sum_{s=1}^{n}\log(1+\rho N_s)$$

This minimization problem has no closed-form solution and must be performed numerically.

Note that the computations in steps 1 and 2 are made independently across shape coordinates.

Appendix 3: Changepoint Model

Calculation of the MLE parameters for the linear mixed-effects onset model: Via the Heaviside function, the model implies a sharp changepoint from atrophy rate $y = \alpha + \alpha' a$ in the control to $= \alpha + (\alpha' + \beta')a$ at age $a = t - \Delta$ in the preclinical group, i.e., Δ years before the clinical onset, if the latter is finite. There is no change for control, since their onset time is infinite. We can interpret as the anatomical phenotype changepoint time. The null hypothesis is $\beta_v' = 0$.

In this model, the structural onset time is the same for all shape coordinates. One can relax this assumption by using a heterogeneous onset model in the non null-hypothesis in which the onset time would be indexed across the shape Δ_v.

Estimation Procedure: Start with homogeneous onset. We want to minimize

$$\sum_{v,j,s}\left(y_{vj}(s) - \alpha_v - \alpha_v' a_j(s) - \beta_v' a_j(s) H\left(a_j(s) - \left(t_{symptom} - \Delta\right)\right)\right)^2$$

with respect to the model parameters. We use an alternating minimization procedure that loops over the following steps until stabilization.

1. Use linear least-square regression to generate estimates $\hat{\alpha}, \hat{\alpha}', \hat{\beta}, \hat{\beta}', \hat{\delta}$ for all q's and for fixed value Δ.
2. Let $RSS(q, \Delta)$ denote the residual sum of squares and $L(\Delta)$ denote the log likelihood:
$$RSS(q,\Delta) = \sum_{v,j,s}\left(y_{vj}(s) - \hat{\alpha}_v - \hat{\alpha}_v' a_j(s) - \hat{\beta}_v' a_j(s) H\left(a_j(s) - \left(t_{symptom} - \Delta\right)\right)\right)^2.$$
3. Maximize L(Δ)
$$\hat{\Delta} \text{ B} \arg\max_{\Delta}\left(2L(\Delta) = \text{cst} - n_{subj}\sum_{v}\log\left(RSS(v,\Delta)\right)\right)$$

For the heterogeneous onset model, the second step is simply replaced by the maximization of $2L(v,\Delta) = \text{cst} - n_{subj}\sum_v \log\left(RSS(v,\Delta)\right)$ with respect to Δ (independently for each v) to obtain Δ_q.

The maximization in Δ is made, in both cases, by computing $L(\Delta)$ (or $L(v,\Delta)$) for all Δ over a discrete time interval.

Tests for Significance: The test statistic is the log-likelihood difference between the null hypothesis $H_0^v: \beta_v' = 0$ and the general hypothesis H_1^v, namely $S_v = L_{H_1^v} - L_{H_0^v}$.

The global statistic is then defined by $S^* = \max_v S_v$. P-values are computed using permutation sampling run until a 10% accuracy is reached with high probability. Permutations affect the value of the onset time by expressing t_k as $t_k = a_1(k) + \delta_k$, where $a_1(k)$ is the age of subject k at the beginning of the study (first scan), and permuting the values of δ_k, so that, for a permutation π, the permuted times are $t_k^\pi = a_{1(k)} + \delta_{\pi(k)}$.

A global p-value is obtained as the fraction of permutations π for which the resulting statistic, say S_π^*, is larger than the observed one S^*. When using the heterogeneous onset model, variables v for which S_v is larger than the 95th percentile of the values of S_π^* that were observed via permutations are considered as significant.

Appendix 4:

Here the subfields of the entorhinal cortex (ERC) are summarized. For details see Krimer et al. (1997).

1. Prorhinal (Pr): occupies the most rostral subarea of the ERC. The Pr first appears a few millimeters rostral to the amygdala, but for the most part lies adjacent to it.
2. Lateral (L): Caudally, L replaces Pr cortex. L is bordered dorsally by Pr, and more caudally by I. Ventrally, it is bounded by S.
3. Intermediate (I) has a rostral and caudal subdivision. A third dorso-medial component designated as I superior (Is) is also defined. This region is medial to the intrarhinal sulcus.
4. Intermediate-Rostral (Ir) appears first dorsal to L and then, as it progresses caudally, it extends laterally and borders S.
5. Intermediate-Caudal (Ic) replaces the larger Ir dorso-caudally and abuts the parasubiculum of the hippocampal formation medially and dorsally. Laterally, Ic extends down to adjoin S.
6. Intermediate-Superior (Is) occupies the most medial and superior portion of the entorhinal region, above the intrarhinal sulcus.
7. Medial-Rostral (Mr) Continuing caudally, Mr replaces Ic, bordering the parasubiculum dorso-medially and S ventro-laterally.
8. Medial-Caudal (Mc) This subdivision replaces Mr and merges caudally with the parahippocampal gyrus. Mc is bounded dorso-medially by the parasubiculum and ventro-laterally by S.
9. Sulcal (S) is a subdivision between the medial subdivisions of Pr, L, I, M. It extends onto the medial bank of the collateral sulcus, and borders obliquely with the neighboring perirhinal cortex.
